# Studies towards hcTnI Immunodetection Using Electrochemical Approaches Based on Magnetic Microbeads

**DOI:** 10.3390/s18082457

**Published:** 2018-07-29

**Authors:** Alejandro Hernández-Albors, Gloria Colom, J.-Pablo Salvador, M.-Pilar Marco

**Affiliations:** 1 Nanobiotechnology for Diagnostics (Nb4D), Department of Chemical and Biomolecular Nanotechnology, Institute for Advanced Chemistry of Catalonia (IQAC) of the Spanish Council for Scientific Research (CSIC), Jordi Girona 18-26, 08034 Barcelona, Spain; ahernandezalbors@gmail.com (A.H.-A.); gloria_colom@hotmail.com (G.C.); pilar.marco@cid.csic.es (M.-P.M.); 2 CIBER de Bioingeniería, Biomateriales y Nanomedicina (CIBER-BBN), Jordi Girona 18-26, 08034 Barcelona, Spain

**Keywords:** human cardiac troponin I, magnetic beads, magnetoimmunosensor, cadmium quantum dots, Streptavidin-Horseradish Peroxidase

## Abstract

Different electrochemical strategies based on the use of magnetic beads are described in this work for the detection of human cardiac troponin I (hcTnI). hcTnI is also known as the gold standard for acute myocardial infarction (AMI) diagnosis according to the different guidelines from the European Society of Cardiology (ESC) and the American College of Cardiology (ACC). Amperometric and voltamperometric sandwich magnetoimmunoassays were developed by biofunctionalization of paramagnetic beads with specific antibodies. These bioconjugates were combined with biotinylated antibodies as detection antibodies, with the aim of testing different electrochemical transduction principles. Streptavidin labeled with horseradish peroxidase was used for the amperometric magnetoimmunoassay, reaching a detectability of 0.005 ± 0.002 µg mL^−1^ in 30 min. Cadmium quantum dots-streptavidin bioconjugates were used in the case of the voltamperometric immunosensor reaching a detectability of 0.023 ± 0.014 µg mL^−1^.

## 1. Introduction

Cardiovascular diseases (CVDs) represent one of the leading causes of death globally. According to the World Health Organization (WHO) in 2012, 17.5 million deaths were caused by these types of pathologies, and more than 80% of these deaths were due to heart attacks and strokes [1]. There are indications that the number of deaths by CVDs will keep rising, and by 2030, it is estimated that almost 23.6 million people will die because of these diseases.

The early and quick diagnosis of CVDs is very important, not only for health and patient survival, but also for cost and time efficiency in a successful diagnosis and prognosis of the illness. Traditionally, AMI diagnosis has been based on the WHO criteria, whereby patients must meet at least two out of three conditions: Ischemia symptoms such as the characteristic chest pain, significant changes in the diagnostic electrocardiogram (ECG), and elevations of the concentration in blood of the creatine kinase (CK-MB) biomarker [2]. Despite ECG being an important tool for diagnosing and monitoring the disease [3,4,5], it is not a good confirmatory test to diagnose CVDs because, as it is described, around half of the patients with a severe cardiopathy do not show relevant variations in their ECG. However, most of these patients show elevations on the troponin concentration, revealing cardiac muscle necrosis [6], highlighting that an ECG test is not enough to make an early and accurate diagnosis of CVDs [7]. In addition, creatine kinase lacks cardiac tissue specificity and release kinetics give less information about the dimensions of the damage in comparison with other biomarkers [8]. For all these reasons, diagnosis criteria for AMI were modified in 2000 by the European Society of Cardiology (ESC) and the American College of Cardiology (ACC), replacing CK-MB biomarker by human cardiac troponin I (hcTnI) as the gold biomarker for the assessment of cardiac damage [8,9]. After an AMI episode, free hcTnI is released to the bloodstream indicating necrosis of the cardiac muscle and increasing the mortality risk, even at concentrations below 0.06 ng mL^−1^. For this reason, current guidelines suggest the use of the 99th percentile of troponin concentration from a healthy reference population as a cutoff for any tool focused in the diagnostic and prognostic of the cardiac illness [10,11,12,13]. In this context, different immunological methodologies have been developed to detect hcTnI in human samples. Electrochemiluminiscent immunoassays (ECLIA) [14,15,16], Fluoroimmunoassays [16,17,18,19,20], and enzyme-linked immunosorbent assay (ELISA) [21,22,23,24] have been widely used for AMI diagnosis. However, these techniques require expensive laboratory equipment, well-trained personnel and are time-consuming techniques, especially taking into account that a timely and reliable diagnosis is required for appropriate patient treatment.

In this sense, immunosensors based on electrochemical transduction have been considered an effective analytical approach, particularly because of their accuracy, high sensitivity, simplicity, low cost and short response time, reaching in some cases low limits of detection [25,26,27,28,29]. Specifically, electrochemical immunosensors based in the use of magnetic beads have improved the analytical performance of the immunoassays due to the high surface-area-to-volume ratio of the particles, conferring higher probability of interaction between the target analyte and the bioreceptor immobilized onto the beads surface and, consequently, achieving faster assays kinetics [30,31,32]. Within electrochemical immunosensors, techniques can be found based on impedance spectroscopy, which allows label-free and real-time detection measuring small changes in the surface of the electrode. Accordingly, other works are described in the literature based on the use of functionalized magnetic beads in combination with impedance spectroscopy, reaching appropriate values of limit of detection regarding clinical guidelines [31,33,34,35,36,37].

In this work, different approaches based on the use of the magnetic beads-antibody bioconjugates targeting hcTnI have been developed as a proof of concept. Magnetic beads were combined successfully with materials from different fields, while avoiding non-desirable interactions, and reducing, in some cases, the assay time and setting the stage for the development of multiplexed electrochemical immunosensors based on the use of the magnetic beads. Furthermore, the electrochemical immunosensors described in this text were compared in terms of assay time, background noise, limit of detection and microbead performance.

## 2. Materials and Methods

### 2.1. Chemicals and Biochemicals

Human cardiac troponin I (hcTnI) was purchased from Life Diagnostics Inc. (West Chester, PA, USA), aliquoted and stored at −80 °C until being used. Human skeletal troponin I (hsTnI) was kindly provided by Dr. Tamas Mészáros from Semmelweiss University (Budapest, Hungary), aliquoted and stored at −80 °C until used. Streptavidin-Horseradish Peroxidase bioconjugate (Sigma-Aldrich, St. Louis, MO, USA) was dissolved in 10 mM of phosphate saline buffer (PBS) solution at pH 7.5 at a final concentration of 1 mg mL^−1^, aliquoted and stored at −20 °C until used. EZ Link sulfo-NHS-LC-LC-Biotin reagent was purchased from Pierce Biotechnology (Rockford, IL, USA). *N*-ethyl-*N*-dimethylaminopropyl-carbodiimide (EDC), *N*-hydroxysulfo-succinimide (NHS), bovine serum albumin (BSA), avidin from egg white, casein from bovine milk and 2-(4-Hydroxyphenylazo) benzoic acid (HABA) were purchased to Sigma-Aldrich. Methyl-(octaethyleneglycol) amine (mPEG-Amine) was acquired from Laysan Bio, Inc. (Arab, AL, USA). Other chemicals and biochemicals used were purchased to Sigma Chemical Co. (St. Louis, MO, USA) and all salts were provided by Merck (Darmstadt, Germany). SiMAG-Carboxyl magnetic beads (SiMAG-Carboxyl, 1 µm Ø, Prod. No. 1402) were purchased from Chemicell GmbH (Berlin, Germany). Qdot^TM^ 585 Streptavidin Conjugate (Qdot-SAv) was purchased from Molecular probes® (Eugine, OR, USA).

### 2.2. Materials and Instruments

Electrochemical measurements were carried out using a portable multipotentiostat µSTAT 8000P (DropSens S.L., Llanera, Spain). Screen-printed carbon array electrodes were purchased from DropSens (SPCE, DRP-8X110, working electrode 2.56 mm Ø). Each device displayed eight 3-electrode electrochemical cells, each of them including a carbon-based working electrode, an Ag pseudo-reference electrode and a carbon counter electrode. The magnetic separation during the different washing steps involved in the immunoassay procedure was performed using a 12-tube magnetic separator rack (MagnaRackTM Cat. No. CS15000, Carlsbad, CA, USA,). A polymethylmethacrylate support (PMMA support) with 8 embedded neodymium magnets, designed and manufactured by Micro-Nano Technologies Unit, of the Unique Scientific and Technical Infrastructures (U8 of the ICTS “NANBIOSIS”) from Institute of Microelectronics of Barcelona (IMB-CNM, Barcelona, Spain), was used to immobilize the modified magnetic beads onto the surface of each of the eight SPCE to perform electrochemical measurements. An IKA MS 3 digital shaker (IKA^®^-Werke GmbH & Co. KG, Staufen, Germany) was used at 700 rpm to incubate magnetic beads at the different stages of each of the immunoassays. The pH and the conductivity of all buffers and solutions were measured with a pH-meter pH 540 GLP and a conductimeter LF 340, respectively (WTW, Weilheim, Germany). Round bottom, non-treated plates were purchased from Nirco (Barberà del Vallés, Spain). Polystyrene Immulon 2 HB™ and MaxiSorp™ microtiter plates were purchased from Nunc (Roskilde, Denmark). Washing steps were carried out using a SLY96 PW microplate washer (SLT Labinstruments GmbH, Salzburg, Austria). Dilution plates were purchased from Nirco (Barberà del Vallés, Spain). Absorbances were read on a SpectramaxPlus (Molecular Devices, Sunnyvale, CA, USA). The electrochemical data obtained was analyzed using DropView 8400 software (DropSens S.L., Llanera, Spain). The calibration curves and different fittings were analyzed using GraphPad Prims 5.03 (GraphPad Software Inc., San Diego, CA, USA).

### 2.3. Buffers and Solutions

PBS was 0.01 mol L^−1^ phosphate buffer, 0.14 mol L^−1^ in NaCl and 0.003 mol L^−1^ in KCl saline solution at pH 7.5. PBST was PBS with 0.05% (*v*/*v*) Tween 20. PBST-Casein was PBST with 0.15% (*w*/*v*) casein. Coating buffer was 0.05 M carbonate-bicarbonate buffer, pH 9.6. Citrate buffer was a 0.04 M solution of sodium citrate, pH 5.5. The substrate solution for optical measurement was 0.01% TMB (3,3′,5,5′-tetramethylbenzidine) and 0.004% H_2_O_2_ in citrate buffer. For amperometric measurements, citrate buffer-KCl was prepared with citrate buffer containing 0.1 mol L^−1^ KCl with 0.001% TMB and 0.0004% H_2_O_2_. For voltamperometric measurements, an acetate buffer was composed by 0.5 M sodium acetate buffer pH 5.5 and 1 µg mL^−1^ Bi (III).

### 2.4. Polyclonal Antibody Production against hcTnI

Antibodies As220 and As221 were raised by immunization of native human cardiac troponin I (hcTnI). Two female New Zealand white rabbits, weighing 1 to 2 kg each, were immunized following the immunizing protocol already described [38]. The evolution of the immunization was followed by titration assays by measuring the binding of a serial dilutions of the antisera to a microplate coated with hcTnI. After an acceptable antibody titer was observed, the animals were exsanguinated, and the blood was collected in vacutainer tubes provided with a serum separation gel. Antisera were obtained after centrifugation step and stored at −80 °C in the presence of 0.02% NaN_3_. Polyclonal antibodies, pAb220 and pAb221 were purified for further bioconjugation procedures, first by ammonium sulphate precipitation and then by protein A affinity chromatography [39].

The production of the antibodies was performed with the support of the U2 of the ICTS “NANBIOSIS”, more specifically by the Custom Antibody Service (CAbS, CIBER-BBN, IQAC-CSIC).

### 2.5. Preparation of Biotinylated Antibody Bioconjugates

Biotin-labeled antibodies were prepared from purified pAb220 and pAb221 and coupled to an EZ Link sulfo-NHS-LC-LC-Biotin according to the specifications provided by the supplier, but with slight modifications. Briefly, 2 mg of each antibody were dissolved in 1 mL of PBS. Then, 27 µL of 10 mM of EZ-Link sulfo NHS-LC-LC-Biotin solution (7 mg/mL of ultrapure water) were added to each antibody drop by drop under continuous stirring for 1 h. The labeled antibodies were purified by dialysis and stored freeze-dried at −80 °C. Working aliquots at 1 mg mL^−1^ were prepared in PBS and stored at −20 °C. Biotinylated antibodies pAb220-B and pAb221-B were characterized following HABA/Avidin procedure according to the supplier protocol. In all cases, the bioconjugates showed between 3 and 4 biotin moieties per antibody.

### 2.6. Sandwich ELISA for cTnI

The calibration curve for hcTnI using As220 and pAb221-B antibodies was developed as follows. Immulon 2 HB™ microtiter plates were coated with the capture antiserum As220 (1/16000 dilution in coating buffer, 100 µL/well), stored overnight at 4 °C and covered with adhesive plate sealers. After this time, microplates were washed four times in 300 µL/well with PBST. hcTnI standard solutions (from 0 to 200 ng mL^−1)^ were prepared in PBST-Casein and added to the microtiter plates, 100 µL/well. After 30 min, the plates were washed again and a solution of the biotinylated antibody pAb221-B (2.5 µg mL^−1^ in PBST, 100 µL/well) was added to the plates. After 30 min and another washing step, a solution of Streptavidin-Horseradish Peroxidase bioconjugate (SAv-HRP) was added to the microplates (0.17 µg mL^−1^ in PBST, 100 µL/well). After 30 min, the plates were washed again, and the substrate solution was added (100 µL/well, protected from light). Color development was stopped after 30 min by adding 4 N H_2_SO_4_ (50 µL/well), and the absorbances were read at 450 nm. The standard curve was fitted to a linear regression. In this case, the limit of detection (LOD) was established as the analyte concentration corresponding to the sample blank value plus three standard deviations. In the same way, the limit of quantification (LOQ) was established as the analyte concentration corresponding to the sample blank value plus ten standard deviations.

### 2.7. Specificity Studies

Specificity studies for both antibodies were carried out by following the sandwich ELISA procedure previously described. In this case, two different calibration curves were performed in PBST-Casein buffer, one for hcTnI and the other for the skeletal isoform, hsTnI (from 0 to 250 ng mL^−1^, 100 µ/well). The standard curve was fitted to a linear regression, and the slopes for each of the fittings were compared to assess the specificity of the antibodies produced against hcTnI.

### 2.8. Preparation of the Magnetic Beads-Antibody Bioconjugates

The magnetic microbeads-antibody bioconjugate (MB-pAb220) was prepared by covalent immobilization to magnetic microbeads with a purified fraction of pAb220 immunoglobulins (described above). Briefly, 10 mg of microbeads were washed twice with 0.1 M MES pH 5.0 buffer solution and resuspended in 250 µL of a solution of 1-ethyl-3-(3′-dimethylaminopropyl) carbodiimide (EDC, 10 mg, 0.05 mmol) prepared in MES buffer, to activate carboxylic groups. The final mixture was gently shaken for 15 min at RT. After the activation step, microbeads were washed twice with MES buffer and resuspended in 250 µL of PBS solution containing 50 µg of pAb220 antibody and incubated for two hours at RT with gentle shaking to avoid microbeads aggregation. After the coupling step, the supernatant was removed and kept for further conjugation yield quantification. Then, the beads were washed three times with PBS and resuspended in 250 µL of 10 mM PBS pH 7.5 with 1% (*w*/*v*) of mPEG-NH_2_ buffer and incubated at RT overnight with gentle stirring to block unreacted sites of the microparticles. Finally, after a washing step, beads were resuspended and put into storage in 250 µL of 10 mM PBS pH 7.5 with 0.5% (*w*/*v*) of mPEG-NH_2_ buffer.

### 2.9. Magneto-ELISA (mELISA) Protocol

The calibration curve for hcTnI detection using MB-pAb220 bioconjugate was developed as follows. All steps were performed in a round-bottom 96 microplate without any surface treatment. After each incubation step, the magnetic beads were washed by placing the microplate on the magnetic rack, allowing the beads to migrate to the magnet until the liquid was clear (approximately 1 min) and then removing the supernatant. hcTnI standards solutions (from 0 to 250 ng mL^−1^ in PBST, 100 µL) were placed in the microplate wells and mixed with a solution of MB-pAb220 (0.1 mg mL^−1^ in PBST, 0.01 mg of beads/well, 100 µL/well). After 30 min of incubation at RT with gently stirring, microbeads were washed with PBST (400 µL, 3 times) and resuspended in a solution of the pAb221-B conjugate (0.5 µg mL^−1^ in PBST, 100 µL/well). After 30 min of incubation under the same conditions described before, magnetic beads were washed again and SAv-HRP was added (0.17 µg mL^−1^ in PBST, 100 µL) for 30 min at RT. Then, beads were washed, and the substrate solution was added and incubated again for 30 min at RT. The enzymatic reaction was stopped by adding 4 N H_2_SO_4_ (50 µL). Finally, supernatants were removed from the magnetic beads by magnetic separation and placed in other microplate for measuring absorbance at 450 nm. The standard curve was fitted to a linear regression.

### 2.10. Amperometric Magneto Immunosensor (AMIS) Protocol

**Figure 1:**
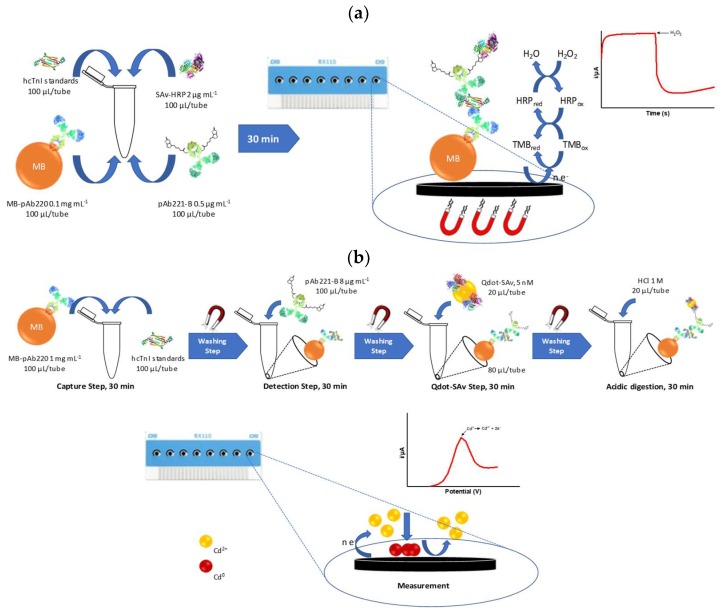
(**a**) Scheme of AMIS procedure. After all the biorecognition steps, the magnetic beads complex (MB-complex) was resuspended and immobilized onto the surface of the working electrode. Then, current intensity increased after substrate addition proportionally to the concentration of the hcTnI in the sample. (**b**) Scheme of the VMIS procedure. After all the biorecognition steps, the MB-complex was digested under acidic conditions provoking cadmium release from Qdot-SAv bioconjugates. Cadmium ions were placed in the electrochemical cell by magnetic rack separation. Then, deposition voltage was applied, and intensity of the peak was reached by square wave voltammetry (SWV).

All steps were performed in 2 mL safe-lock tubes. After each incubation step, magnetic beads were washed and supernatants were removed by magnetic separation rack until the beads had migrated to the magnet and the liquid was clear (approximately 1 min). hcTnI standards solutions (from 0 to 125 ng mL^−1^ in PBST, 100 µL) were placed in the tubes and mixed with a solution of MB-pAb220 (0.1 mg mL^−1^ in PBST, 0.01 mg of beads/tube, 100 µL/tube), previously washed 3 times with PBST. Then, a solution of pAb221-B (0.5 µg mL^−1^ in PBST, 100 µL/tube) and a solution of of SAv-HRP (2 µg mL^−1^ in PBST, 100 µL/tube) were immediately added to the tubes with the hcTnI standards and the microbeads. The mixture was incubated for 30 min at RT with gentle stirring. Afterwards, magnetic beads were washed with PBST (3 × 800 µL), resuspended in citrate-KCl buffer (100 µL) and captured onto the surface of the working electrode using a magnet located under the SPCEs. Finally, amperometric measurements were carried out at an applied potential of −0.10 V vs. Ag pseudo-reference electrode. After current stabilization, 10 µL of substrate solution prepared in citrate buffer-KCl was added and current was recorded once again after its stabilization. The standard curve was fitted to a linear regression. A schematic representation of the whole procedure is shown in Figure 1a.

### 2.11. Voltamperometric Magneto Immunosensor (VMIS) Protocol

All the immunochemical steps were performed in 2 mL safe-lock tubes, and all the quantities referred to in this procedure are the amount added per tube. After each incubation step, magnetic beads were washed (3 × 800 µL, PBST) by magnetic separation, placing the tubes in a magnetic rack (approximately, 1 min), as described above. hcTnI standard solutions were prepared as previously described (from 0 to 250 ng mL^−1^ in PBST, 100 µL) and mixed with a solution of the MB-pAb220 (1 mg mL^−1^ in PBST, 0.1 mg of beads/tube, 100 µL). After 30 min of incubation at RT and gently stirring, magnetic beads were washed and resuspended in a solution of pAb221-B at 8 µg mL^−1^ (100 µL, PBST). After 30 min of incubation at RT and gently stirring, the beads were washed again as previously described and resuspended in 80 µL of PBST. After a complete resuspension of the microbeads, 20 µL of a solution of 5 nM of Qdot-SAv prepared in PBST was added to each tube and incubated at RT for 30 min with gently stirring. After this time, beads were washed and Cd^2+^ ions were released by acidic digestion adding 20 µL of 1 M HCl for 30 min at RT with gentle stirring. Afterwards, each tube was placed onto the magnetic rack and supernatants were placed onto the surface of the working electrodes, each of them containing 40 µL of acetate buffer. Square Wave Voltammetry (SWV) was used to perform the electrochemical measurements using Ag as a pseudo reference electrode. A condition voltage (0.6 V, 60 s) was initially applied, followed by a deposition voltage (−1.4 V, 180 s). After a 15 s rest period, anodic stripping voltammetry was performed (from −1.20 to −0.6 V, step voltage of 10 mV, amplitude 50 mV and frequency of 20 Hz). Under these conditions, well-defined stripping current peaks at approximately −0.85 V (oxidation/stripped voltage) were obtained. A schematic representation of the whole procedure is shown in Figure 1b. The height of the peak (current), proportionally to the concentration of the hcTnI in each sample, was calculated using DropView 8400 software. Standard curves were obtained plotting the current versus the hcTnI concentration and fitting the points to the linear regression.

## 3. Results and Discussion

### 3.1. ELISA Sandwich for hcTnI

**Figure 2:**
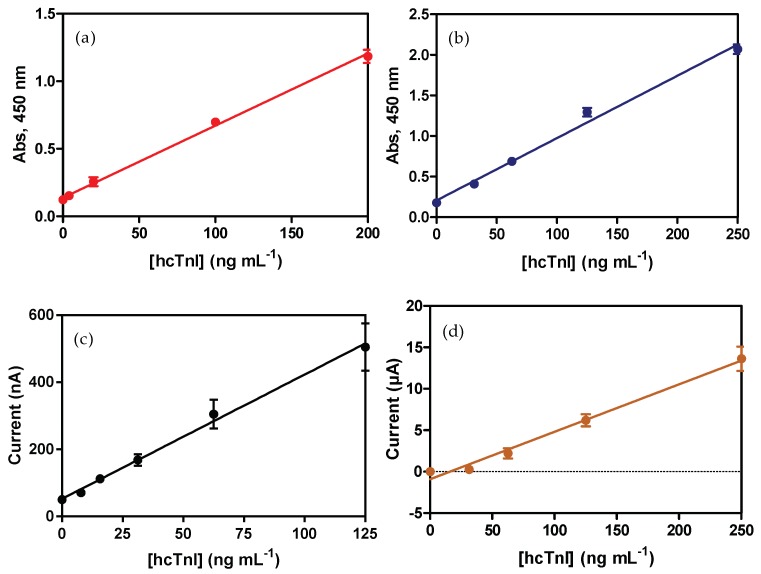
Calibration curves for the different immunoassays developed for the detection of hcTnI. (**a**) ELISA calibration curve for hcTnI in buffer using the immunoreagent produced. As220 was used as a capture antibody at a dilution of 1/16,000, and pAb221-B at 2.5 µg mL^−1^. Each point was the average of at least two-well replicates, and the assay was run on two different days. (**b**) mELISA calibration curve for hcTnI in buffer using the different bioconjugates prepared as described previously. MB-pAb220 was used as a capture probe at 0.1 mg mL^−1^ and pAb221-B as a detection antibody at a concentration of 0.5 µg mL^−1^. SAv-HRP was used at a concentration of 0.17 µg mL^−1^. Each point was the average of at least two well replicates, and the assay was run on two different days. (**c**) AMIS calibration curve for hcTnI detection in buffer. MB-pAb220 was used as a capture probe at 0.1 mg mL^−1^ and pAb221-B as a detection antibody at a concentration of 0.5 µg mL^−1^. SAv-HRP was used at a concentration of 2 µg mL^−1^. Each point was the average of at least three well replicates, and the assay was run on three different days. (**d**) VMIS calibration curve for hcTnI detection in buffer using the immunoreagents produced and after different optimization steps. MB-pAb220 was used as a capture probe at 1.0 mg mL^−1^ and pAb221-B as a detection antibody at a concentration of 8 µg mL^−1^. Qdot-SAv was used at a concentration of 5 nM. Each point was the average of at least three well replicates, and the assay was run on three different days.

After preparation of the pAb-Biotin bioconjugates, all antibody combinations were evaluated by sandwich ELISA using As220/As221 as the capture antibody, together with pAb221-B/pAb220-B as the detection antibody. The initial criterion followed to select the best combination was the signal-to-noise ratio (S/N ratio). The highest S/N ratio observed in all the combinations was when As220 was used as the capture probe, regardless of the pAb-biontin used. The pair formed by As220 as the capture antibody and pAb221-B as the detection antibody showed the highest signal and the lowest background noise (data not shown). This combination was selected to develop further immunoassays. According to the non-specific adsorption observed for the hcTnI protein to the surface of the microplate, further steps were carried out in order to minimize the background noise. This non-desired adsorption was because of its high isoelectric point of hcTnI (pI = 9.98) and its high hydrophilic behavior [40]. Thus, different microplates with different surface treatment were evaluated along with different proteins as additives to avoid the non-specific adsorption. Finally, Immulon 2 HB™ microplates, together with casein as an additive, showed a significant decrease of the background noise and were therefore chosen to develop the calibration curve (see Supplementary Materials, Figure S1a,b). The decrease in the background noise could be explained in terms of the decreased affinity for hydrophilic molecules shown by Immulon 2 HB™ microplates in contrast to MaxiSorp™ microplates. On the other hand, due to its small size (19 to 25 kDa, depending on the casein subunit), casein can occupy empty spaces of the microplate, blocking non-specific adsorptions more efficiently [37,38] than other typical blocking proteins, such as BSA. In this case, adding casein together with hcTnI in the same buffer significantly decreases the high background noise, avoiding an extra blocking step in the ELISA procedure. Under these conditions, calibration curves in ELISA sandwich format were developed reaching a detectability value of 0.010 ± 0.002 µg mL^−1^ for hcTnI (Figure 2a and Table 1).

### 3.2. Specificity Studies

To prove the specificity of the antibodies produced against hcTnI, calibration curves for hcTnI and its skeletal isoform, skeletal human troponin I (hsTnI), were performed in ELISA. Both curves were fitted to a linear regression (See Supplementary Material, Figure S2). The slope of both immunoassays was compared in order to determine the affinity and the sensitivity of the antibodies against hsTnI and, as shown in the range of concentration evaluated, no affinity of As220 and As221 against skeletal troponin isoform was observed, suggesting that both antibodies were able to distinguish between two proteins because of their high specificity against their antigen.

### 3.3. Assessment of the Bioactivity of Magnetic Beads-Antibody Bioconjugates

One of the main advantages of using magnetic beads-antibody bioconjugates is their high surface-to-volume ratio, which confers a higher probability of interaction with the analyte while increasing the performance of the assay, promoting the kinetics, and the possibility of increasing the efficiency of the isolation of the analyte from the matrix due to their superparamagnetic properties [41]. First, the efficiency of the antibody coupling onto the microbeads surface was assessed by evaluation of the supernatant after the coupling step. The amount of antibody not immobilized was quantified by a Bio-Rad protein Assay, reaching in all cases a conjugation yield of 95 ± 4%. Further experiments were focused in the assessment of the functionality of the antibodies by mELISA. First, the concentration of each of the immunoreagents (MB-pAb220 and pAb221-B) was optimized. The concentration of each of the antibody bioconjugates was selected according to the S/N ratio. In this sense, two different concentrations of MB-pAb220 were evaluated against two concentrations of hcTnI (0 and 1 µg mL^−1^), together with three different concentrations of the pAb221-Biotin bioconjugate (2, 1 and 0.5 µg mL^−1^). Concentrations of 0.1 mg mL^−1^ of MB-pAb220 and 0.5 µg mL^−1^ of the biotinylated antibody were selected to perform mELISA and amperometric immunosensor given the highest S/N ratio shown (see Supplementary Material Figure S3). In this case, due to the characteristics of the beads surface, casein addition to the assay buffer was not necessary to avoid non-specific adsorption.

Finally, magnetic bead bioconjugates were characterized by developing a calibration curve followed by colorimetric transduction. The limit of detection shown by mELISA prior to the optimization was in the same order in comparison with LOD showed by ELISA, 0.023 ± 0.001 µg mL^−1^ and 0.010 ± 0.002 µg mL^−1^, respectively (see Figure 2a–b and Table 1).

### 3.4. Development of Electrochemical Immunosensors for hcTnI Detection

The two electrochemical immunosensors presented here are based on the use of magnetic beads modified with specific antibodies against hcTnI as a selective capture probe in a fast and reliable way. Two different electrochemical transductions were investigated and compared in terms of microbead features, background noise and analytical performance, among others.

#### 3.4.1. Amperometric Magneto-Immunosensor (AMIS)

The Amperometric Magneto-Immunosensor (AMIS) is based on the registration of the current intensity produced by the enzymatic reaction catalyzed by the Horseradish-Peroxidase (HRP) present in the SAv-HRP bioconjugate when a specific substrate is used. In this case, HRP can oxidize the substrate (H_2_O_2_ to H_2_O) leading to TMB oxidation, and subsequently reduced again by the electrode applied potential (Figure 1a). The acquired current intensity is directly proportional to the amount of the HRP immobilized onto the MB-complex and directly correlated with the concentration of hcTnI in the sample.

Using magnetic bead bioconjugates confers the possibility of reusing the surface of the electrode; due to the electrode, surface modification is not needed to perform measurements. In this regard, electrodes were evaluated after each experiment observing the background current in the absence of beads and compared with the electrochemical current of a control electrode for which no experiment was carried out. In this sense, the background current started to be significantly different after the third electrochemical measurement.

For amperometric transduction, the concentrations of all of the immunoreagents, MB-pAb220, pAb221-B and SAv-HRP, as well as their incubation times, were previously optimized by mELISA. With the main objective of significantly reducing the assay time and accomplishing the clinical guidelines recommendations, incubation time was optimized, evaluating the response of the immunoassay in terms of maximum signal and background noise when only a single step was performed, instead of a sequential-step assay. All the immunoreactants were mixed at the concentrations previously optimized by mELISA, and after 30 min of incubation time, colorimetric readout was achieved by adding the corresponding substrate (see Supplementary Materials, Figure S4a). As can be seen, the maximum response of the immunoassay in presence of hcTnI decreased significantly when the immunoassay was performed in a single step. However, further optimization of the concentration of the SAv-HRP led us to improve the S/N ratio working at 2 µg mL^−1^ (see Supplementary Materials, Figure S4b).

Finally, the calibration curve for hcTnI was performed (see Figure 2c) by mixing different standard solutions of the cardiac biomarker with MB-pAb220, pAb221-b and SAv-HRP at optimized concentrations and incubating for 30 min at RT. Electrochemical measurements of the immunocomplexes formed were obtained in the electrochemical cell after capturing the beads and the substrate addition.

Both AMIS and mELISA employed different concentration of magnetic beads according to the different immunochemical protocol. For practical purposes, the amperometric immunosensor was implemented using 2 mL safe-lock tubes. In the case of AMIS, the final concentration of beads was 0.025 mg mL^−1^, while in the case of mELISA, the concentration of beads in the capture step was 0.05 mg mL^−1^, and 0.1 mg mL^−1^ in the remaining steps. Despite this difference, the S/N ratio for both immunoassays at hcTnI level of 125 ng mL^−1^ was 12 ± 3 for AMIS against the 7.3 ± 0.1 for mELISA. These observations could suggest that the beads are more efficient if they are confined in a larger container than a 96-well plate microwell. On the other hand, the non-reversible formation of doublets species when MB-Ab and antigen (Ag) are incubated together, and a magnetic field is subsequently applied, has been well described [42]. In this sense, incubation of all the immunoreagents at the same time, as described in the AMIS procedure, could reduce the possibility of forming MB-Ab-Ag-Ab-MB tandems due to the presence of the detection antibody, thus improving the sensitivity of the immunoassay.

In short, according to the different analytical parameters obtained in ELISA, mELISA and AMIS, electrochemical magnetic bead-based immunosensor performance (see Table 1) not only improves the limit of detection, but also significantly reduces the total assay time.

#### 3.4.2. Voltamperommetric Magneto-Immunosensor (VMIS)

The voltamperometric immunosensor is based on the acquisition of the characteristic oxidation potential from a specific metallic nanoprobe, such as the cadmium-containing commercial Qdot-Streptavidin bioconjugate. These Qdots are composed by a CdSe core encapsulated by a shield of ZnS coated with a commercial polymer with specific functional groups to immobilize streptavidin. After acidic digestion, the ZnS layer is dissolved and cadmium ions are released in the medium. Then, applying a deposition voltage, cadmium ions are reduced onto the surface of the working electrode and oxidized again afterwards by stripping voltammetry, showing a characteristic current peak corresponding to the oxidation potential of the cadmium (Figure 1b). The peak intensity in this case is directly related to the amount of cadmium in the Qdots immobilized by the interaction streptavidin-biotin and then is directly proportional to the hcTnI in the sample.

**Figure 3:**
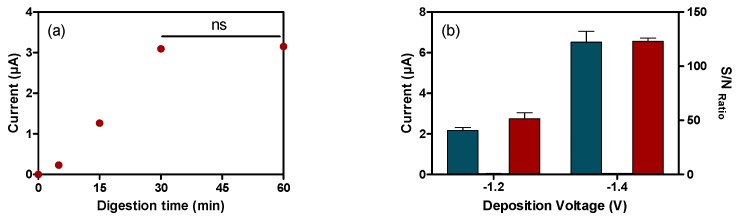

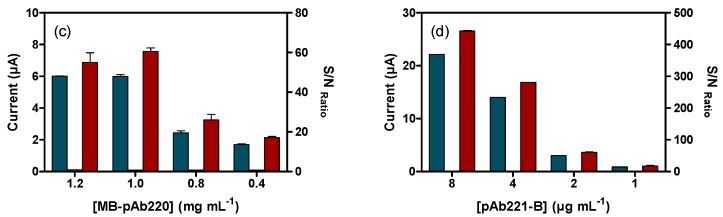
Optimization of the magneto bead-based voltamperometric immunosensor for hcTnI at two different levels (1 µg mL^−1^, blue bar, 0 µg mL^−1^, yellow bar, S/N ratio, red bar) using cadmium Qdot-SAv bioconjugates. (**a**) Digestion time in acidic conditions (1 M HCl) to release maximum amount of cadmium ions. (**b**) Deposition voltage was optimized, achieving a higher signal without any increasing of the background noise. (**c**) Concentration of beads and the effect of (**d**) detection antibody were fixed. Data are representative of two independent experiments (ns = not significant).

Different parameters were optimized before the development of the calibration curve for hcTnI detection-based Voltamperometric Magneto-Immunosensor. First, the incubation times of the Qdot-SAv bioconjugate to release the maximum amount of Cd^2+^ ions under acidic conditions were optimized. As shown in Figure 3a, no differences were observed in the current achieved after 30 min of incubation at RT and gentle stirring. Afterwards, deposition voltage was also optimized. Current signal in presence of hcTnI was significantly improved when the deposition voltage applied was changed from −1.2 to −1.4 V, with no increase in the background noise (see Figure 3b). Finally, concentrations of the different antibody bioconjugates were optimized at two different levels of hcTnI (1 and 0 µg mL^−1^) to observe which combination showed the highest S/N ratio. First, MB-pAb220 concentration was optimized, evaluating a concentration range from 0.4 to 1.2 mg mL^−1^. A saturation point was reached at a concentration of 1.0 mg mL^−1^ of magnetic beads, setting this value for further assays (see Figure 3c). Afterwards, different concentrations of the biotinylated antibody pAb221-B, ranging from 1 to 8 µg mL^−1^, were assessed. As shown in Figure 3d, current values in the presence of hcTnI increased gradually with higher concentrations of pAb221-B, reaching the highest current value at 8 µg mL^−1^ of the detection antibody. In all cases, the current in absence of hcTnI was nearly undetectable, revealing the total absence of nonspecific adsorptions in the immunoassay.

The calibration curve for hcTnI was performed first by mixing biomarker standard solutions (from 0 to 250 ng mL^−1^, PBST) with the established concentration of the MB-pAb220, further steps are indicated in Figure 1b. After acidic digestion of the Qdots, the measurement of the amount of cadmium in the sample was performed in the electrochemical cell PMMA support, applying SWV procedure. As expected, the current signal recorded was, in this case, directly proportional to the concentration of hcTnI in the sample (see Supplementary Material, Figure S5). The limit of the detection achieved with this immunosensor was 0.023 ± 0.014 µg mL^−1^ (see Figure 2d and Table 1).

In principle, using cadmium quantum dots implies a signal amplification and, consequently, an improvement of the analytical performance of the immunoassay. As has been reported in previous works [43,44,45], the sensitivity of the immunosensor could be improved using these probes, due to the number of Cd^2+^ cations released per Qdot particle. However, the VMIS described in this work, shown similar analytical features as the mELISA immunoassays in terms of detectability. As per the number of beads and their concentration in each step, in comparison with AMIS, the number of beads used in the first capture step is significantly higher (0.1 mg in VMIS and 0.01 mg in AMIS), as is the final concentration. Following the same hypothesis stated previously (See Section 3.4.1), an increase in the concentration of the beads does not have to be directly related to an improvement of the sensitivity, but rather the contrary, as shown in this work. For these reasons, further optimization work could make it possible for VMIS to achieve the required limit of detection for hcTnI.

Nevertheless, for this magnetic bead-based immunosensor, in comparison with colorimetric immunoassays and the amperometric magneto immunosensor described here, in addition to the assay time being able to be reduced by optimization assays, could become a confirmatory tool to assess the degree of cardiovascular damage, due to the possibility of multiplexing, in combination with other metallic nanoprobes other than cadmium, as has been proved [36,46] and, consequently, detecting different biomarkers from the same patient, giving more extensive information about the magnitude of the disease.

## 4. Conclusions

Two electrochemical magnetic bead-based immunosensors have been developed, successfully combining a magnetic beads-antibody bioconjugate with other immunoreactants, depending on the electrochemical transduction chosen. In the case of the AMIS, the magnetic beads-antibody bioconjugate (MB-pAb220) was combined with a detection antibody produced, conjugated and characterized in the laboratory (pAb221-B), together with the rest of immunoreagents, and after a time of 30 min and the addition of the corresponding substrate, an electrochemical response directly related to the concentration of the hcTnI biomarker present in the sample was observed. In this sense, the assay time was significantly reduced in comparison with ELISA and mELISA due to the improvement of the kinetics provided by the magnetic beads, therefore fulfilling the clinical requirements in terms of time for the diagnosis of myocardial infarction. This short-time assay makes AMIS a good candidate to be a useful tool, after further optimization steps to improve detection limit in the ED department for establishing a clear diagnosis of acute myocardial infarction. Furthermore, in comparison with the rest of the techniques developed and described in this text, the detection limit achieved with this methodology was improved, approaching the 99th percentile of a healthy reference population, which is considered optimal for all techniques focused on the detection of this biomarker.

Likewise, it allows the combination of bioconjugates of a different nature, such as MB-pAb220, with the commercial bioconjugate of Qdot-Sav, as well as with the bioconjugate pAb221-B. After several optimization steps, it was possible to develop an electrochemical immunosensor for the detection of hcTnI whose potential lies in the possibility of multiplexing through the use of other metallic electrochemical nanoprobes (Pb, Zn…) to detect different biomarkers which, along with hcTnI, can give useful information about the magnitude of the damage and establish a reliable diagnosis [47].

All the work described here reveals the high potential of magnetic beads as well as their great versatility, since they have been successfully combined with bioconjugates of different natures to develop immunochemical techniques based on different principles, such as colorimetric, amperometric and voltamperometric transduction.

## Figures and Tables

**Table 1:** Analytical features and immunoassay characteristics for the different immunochemical approaches developed for the hcTnI detection.

	ELISA ^a^	mELISA ^b^	AMIS ^c^	VMI ^d^
**Beads (mg)**	-	0.01	0.01	0.1
**[MBs] (mg mL^−1^)**	-	0.05	0.025	0.5
**Total Assay Time (min)**	>120	120	30	120
**Slope**	5.34 ± 0.17	7.68 ± 0.311	3.71 ± 0.29	57.26 ± 3.93
**Ordinate**	0.14 ± 0.01	0.207 ± 0.04	52.03 ± 17.19	−0.91 ± 0.51
**LOD (µg mL^−1^)**	0.010 ± 0.002	0.023 ± 0.001	0.005 ± 0.002	0.023 ± 0.014
**LOQ (µg mL^−1^)**	0.031 ± 0.007	0.075 ± 0.044	0.020 ± 0.006	0.068 ± 0.045
**R^2^**	0.990 ± 0.003	0.988 ± 0.01	0.989 ± 0.004	0.977 ± 0.058

^a^ Enzyme-Linked Immunosorbent Assay; ^b^ Magnetic Enzyme-Linked Immunosorbent Assay; ^c^ Amperometric Magneto-Immunosensor; ^d^ Voltamperometric Magneto-Immunosensor.

## Supplementary Materials

The supplementary materials are available online at http://www.mdpi.com/1424-8220/18/8/2457/s1.
