# Supplementary material for: Studies towards hcTnI Immunodetection Using Electrochemical Approaches Based on Magnetic Microbeads
Source: Sensors (Basel). 2018 Jul 29;18(8):2457. doi: 10.3390/s18082457 (PMC6111321; doi:10.3390/s18082457)
Supplement: Supplementary file 1 [file sensors-18-02457-s001.pdf]

Supplementary Materials: The following are available online at www.mdpi.com/link, Figure S1: title, Table S1: title, Video S1: title.

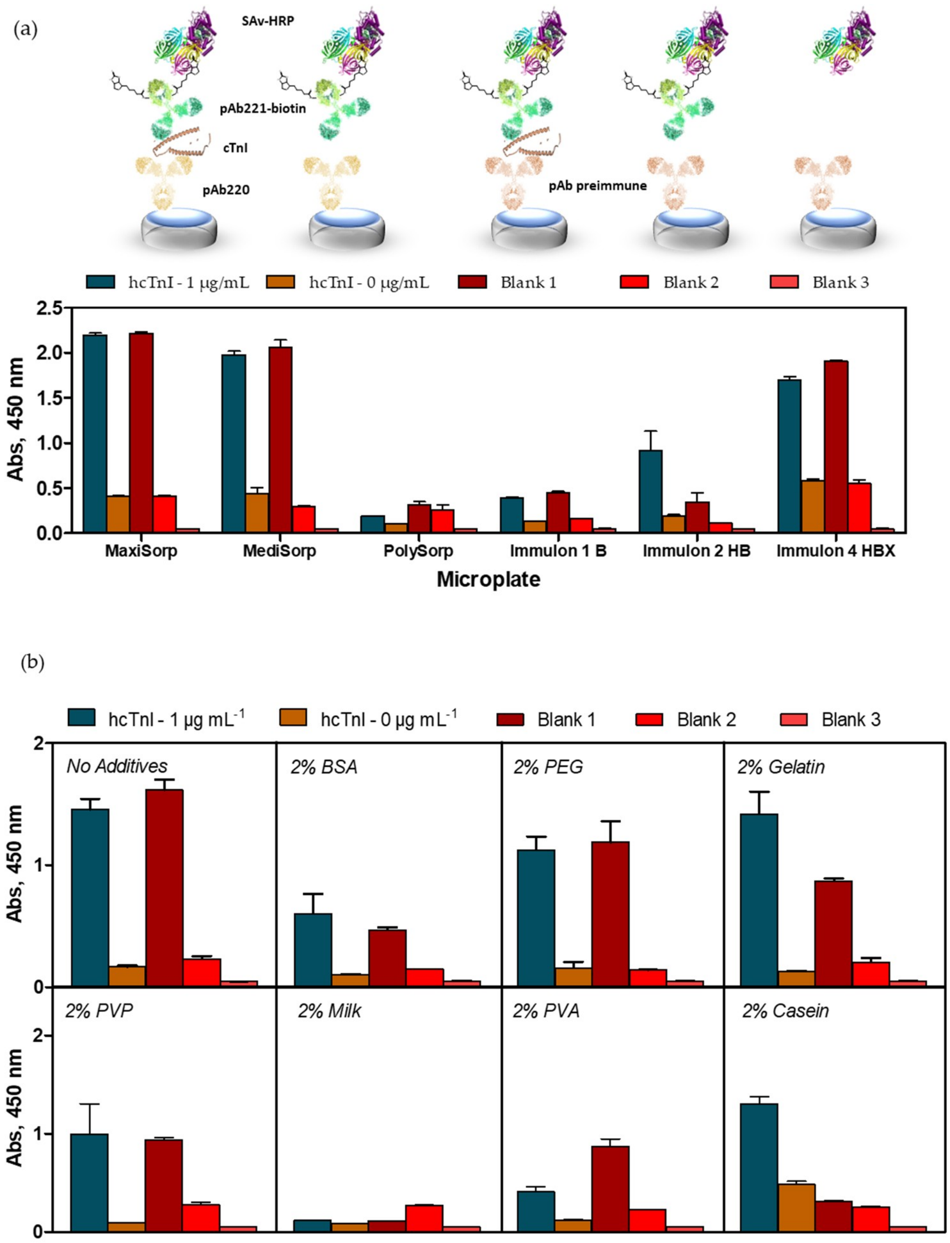

Figure S1. (a) Different controls and blanks are shown to assess the non-specific adsorption of the hcTnI to the polyestirene of the different microplates. Controls with specific antibodies (pAb220 and pAb221-B) with and without hcTnI, together with Blank 01, 02 and 03, done with a non-specific antibody as a capture antibody (Preimmune antibody) were evaluated in all the surfaces. Dark red bare correspond to the signal related to the non-specific adsorption of the biomarker to the microplate. (b) Different proteins and polymers were evaluated as an additive in the hcTnI incubation buffer step (PBS-T with additive) to minimize non-specific adsorption, following the same scheme shown in the Figure S1a and using Immulon 2 HB microplates.

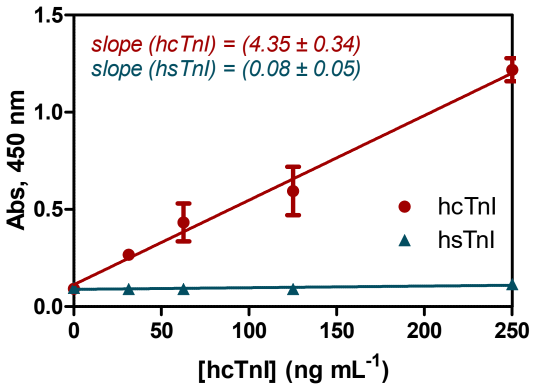

Figure S2. Different calibration curves for hcTnI (red plot) and hsTnI (blue plot) prepared in PBST-Casein buffer. In both cases, As220 was used as a capture antibody at dilution of 1/16000, and pAb221-B at 2.5 µg mL^{-1}. Each point was the average of at least two-well replicates, and the assay was run on a single day.

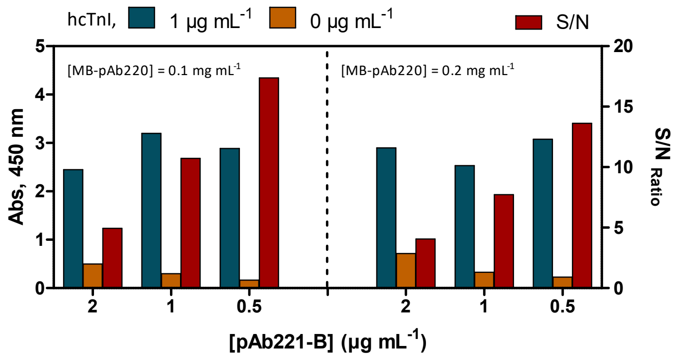

Figure S3. Results from the different optimization assays performed of the bioconjugates MP-pAb220 and pAb221-B. Attending to the signal-to-noise ratio (red bare), calculated as the ratio between absorbance at 1 µg mL^{-1} and the absorbance at 0 µg mL^{-1} of hcTnI, concentration of 0.1 mg mL^{-1} of MB-pAb220 and 0.5 µg mL^{-1} were chosen for develop mELISA and amperometric immunoassay.

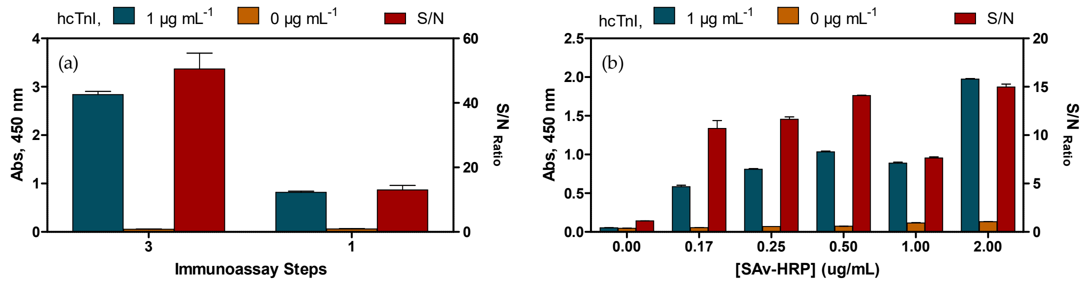

Figure S4. (a) Immunoassay response in presence and absence of hcTnI when it was performed sequentially (3 steps) and in a single step (1 steps). (b) Optimization of the concentration of the SAv-HRP in a single step immunoassay procedure. In all cases, immunoreagents concetration used was, MP-pAb220 at 0.1 mg mL^{-1} and pAb221-B at 0.5 µg mL^{-1}.

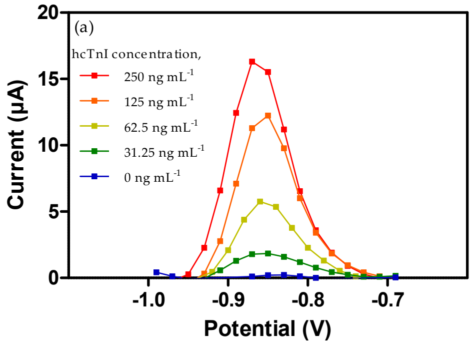

Figure S5. Stripping peaks recorded with the magnetic bead-based voltamperommetric immunosensor measuring different concentrations of hcTnI prepared in buffer. The amplitude of the stripping peak is directly related to the amount of biomarker in the sample.
